# Endpoint stiffness magnitude increases linearly with a stronger power grasp

**DOI:** 10.1038/s41598-019-57267-0

**Published:** 2020-01-15

**Authors:** A. Takagi, G. Xiong, H. Kambara, Y. Koike

**Affiliations:** 1 Tokyo Institute of Technology, Institute of Innovative Research, Yokohama, Japan; 2 Precursory Research for Embryonic Science and Technology (PRESTO), Japan Science and Technology Agency (JST), 4-1-8 Honcho, Kawaguchi, Saitama, 332-0012 Japan

**Keywords:** Neuroscience, Motor control

## Abstract

Humans can increase the endpoint stiffness of their arm to reduce self-generated movement variability and to reject unpredictable perturbations from the environment, like during handheld drilling, thereby increasing movement precision. Existing methods to estimate changes in the endpoint stiffness use robotic interfaces to apply position or force perturbations to measure the arm’s dynamic response. We propose an alternative method of measuring changes in the power grasp force to estimate adaptations in the magnitude of the arm’s endpoint stiffness. To validate our method, we examined how the strength of the power grasp, when holding onto a robotic manipulandum, affected the arm’s endpoint stiffness in three different locations of the workspace. The endpoint stiffness magnitude increased linearly with the grasp force, and this linear relationship did not depend on the arm’s posture or position in the workspace. The endpoint stiffness may have increased as a combination of greater grasp stiffness and greater arm stiffness, since larger co-contraction was observed in the elbow and shoulder with a stronger grasp. Changes in the grasp force could serve as a metric in assessing how humans adapt their endpoint stiffness magnitude.

## Introduction

To fasten a screw into a wall, a screwdriver is guided into the screw’s slotted hole to rotate it. This task is unstable as the screwdriver can slip out of the hole during rotation. Humans cannot react fast enough to correct a slipping screwdriver as visual feedback is delayed by approximately 200 milliseconds. Instead, the central nervous system (CNS) adopts a strategy of co-contracting muscle pairs in a joint to increase the endpoint stiffness of the arm. The endpoint stiffness as defined in this study is the stiffness of the upper arm, including the hand, which generates a restoring force that bring the hand to its equilibrium position. The endpoint stiffness is a mechanical property of the arm, such that the restoring force is generated without sensory feedback delays^1^.

Studies have shown that humans are able to adapt the stiffness of their arm to suppress self-generated motor variability^2,3^ and reject perturbations from the environment^4,5^. Existing methods to estimate the arm’s endpoint stiffness employ a robotic interface to perturb the arm’s position and measure the restoring force generated by the arm^6–9^. Force perturbations can also be employed to estimate the arm’s endpoint stiffness^10^. These methods require a robotic interface, which are expensive and are not portable, making them unsuitable for measurements outside of the laboratory environment. In a recent study, we reported that when subjects had to reach with higher movement precision, they increased their hand’s power grasp force^11^, which we define as the force measured between the palm of the hand and the handle of the robotic interface. The adaptation of the grasp force was similar to the manner in which the arm’s endpoint stiffness has been reported to change in an unstable environment^12^. If the grasp force and the arm’s endpoint stiffness are related, measuring changes in the power grasp force may yield an affordable and portable method of estimating changes in the magnitude of the arm’s endpoint stiffness.

In this study, we deliberately chose to measure the endpoint stiffness by exerting position perturbations through the subject’s hand. Some studies use a mold of the hand that is attached to the handle of the robotic interface to perturb the subject’s arm at the wrist, effectively bypassing the hand and the need to grasp the robotic interface. However, the endpoint stiffness of the arm in real-world tasks does not bypass the hand. When using a hammer or a chisel to carve wood, the endpoint stiffness at the tip of the tool depends on both the stiffness of the grasp and the stiffness of the arm. We define the grasp stiffness to be the stiffness in the connection between the handle of a held object and the hand. The stiffness of the arm arises from the muscular co-contraction of muscles in the elbow and the shoulder. The stiffness of the arm alone may not reflect how humans increase endpoint stiffness in real-world tasks. As such, it is important to ascertain the endpoint stiffness at the hand, i.e. the combined stiffness of the grasp and the arm.

The aim of this study was to assess the relationship between the hand’s power grasp force and the magnitude of the endpoint stiffness of the arm. We hypothesized that this relationship may depend on the arm’s posture, and so we measured the arm’s endpoint stiffness as a function of grasp force in three different postures. When measuring the endpoint stiffness of the arm, we opted for a position perturbation paradigm commonly used in the literature where a hand-held robotic interface perturbs the arm’s position by several millimeters and measures the restoring force to estimate the arm’s endpoint stiffness^8,9,13,14^. We hypothesized that the magnitude of the endpoint stiffness of the arm was positively correlated with the grasp force, and that the posture may interact with this relationship.

## Materials and Methods

### Experimental setup

20 subjects, who all gave their informed consent, participated in the study (10 subjects in the first experiment and 10 subjects in the second experiment). The experimental protocol was approved by the Ethical Review Committee for Epidemiological Studies at the Tokyo Institute of Technology (reference number A17086), and was conducted in accordance with the 1964 Helsinki declaration and its later amendments or comparable ethical standards.

**Figure 1:**
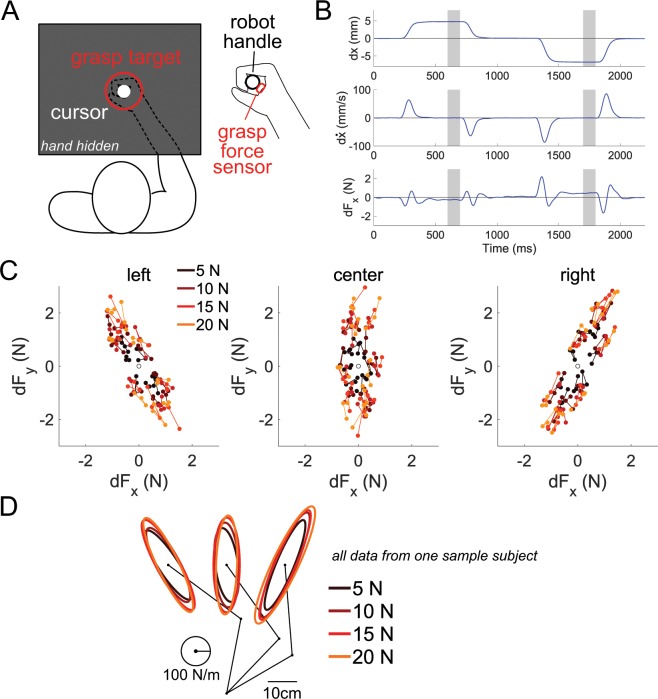
Position perturbations were imposed on the arm to measure its endpoint stiffness as a function of the grasp force. (**A**) Subjects sat in front of the robotic interface and grasped its handle with a three-axis force sensor placed between the palm and the robot’s handle. Subjects had to produce the target grasp force (red circle) by grasping the handle (white circle). Visual feedback of the hand’s position and grasp force were frozen during the position perturbation. (**B**) The temporal profile of the robot’s position, velocity and force along the *x*-axis are shown from a sample subject during two position perturbations. The shaded area shows the 100 millisecond window where the restoring force was measured in response to the position perturbation. The data from this window in the 24 position perturbations per posture per grasp force level was regressed to estimate the arm’s endpoint stiffness for a particular posture and grasp force level. (**C**) The restoring force measured by the force sensor in the left, center and right positions from a sample subject in all trials. The colors denote the restoring force for a specific grasp force. The restoring force was symmetrically distributed around the origin. By linearly regressing this restoring force data with the position perturbation data, the endpoint stiffness was estimated for each grasp force level. (**D**) The endpoint stiffness ellipses are plotted in the left, center and right postures for a sample subject. The color of the ellipse denotes the grasp force level, with lighter traces corresponding to larger grasp force. The size of the ellipse was observed to increase with the grasp force.

The subjects were seated facing the KINARM planar robotic manipulandum from BKIN Technologies (see Fig. 1A). A subject held onto the KINARM interface via a handle that was affixed with a three-axis force sensor (Tec Gihan) to measure the grasp force between the palm of the hand and the handle. The KINARM’s handle itself contains a six-axis force sensor, which was used to measure the arm’s restoring force in the two dimensional plane of the task. An Edero Armon arm support was used under the elbow to support the arm’s weight when using the robotic interface. Visual feedback was provided on a monitor that was placed upside-down such that subjects viewed a reflection of the monitor on a thin film mirror placed above the hand, which obscured it from view. The data from the KINARM and the two force sensors was recorded at 1000 Hz.

Once seated, subjects were instructed to keep their head and torso in the same posture throughout the experiment. This was accomplished by having the subjects rest their forehead on a fixed headrest, with the chest pressing against a table placed below the robotic interface. The position of the shoulder, and the lengths of the forearm and the upper-arm were measured prior to the experiment to determine the posture of the arm.

### Position perturbation

The robotic manipulandum’s position ${\bf{x}}={[x(t),y(t)]}^{T}$, which varied with time *t*, was controlled to maintain its position at a commanded setpoint ${{\bf{x}}}_{0}={[{x}_{0},{y}_{0}]}^{T}$ with a position controller that generated the force

1 $${\bf{F}}={L}_{p}({{\bf{x}}}_{0}-{\bf{x}})-{L}_{d}\dot{{\bf{x}}}$$

where ${L}_{p}=3000$ N/m and *L*_*d*_ = 130 Ns/m.

We measured the arm’s endpoint stiffness in three different positions of the workspace that corresponded to different setpoints x_0_ = [−0.2, 0, 0.2] m, which are hereby referred to as the left, center and right positions, respectively. *y*_0_ = 0.12 m was fixed for all three positions. The joint angles for each posture were ${\theta }_{s}=[81\pm 2,\,49\pm 2,\,35\pm 2]$ degrees and ${\theta }_{e}=[60\pm 3,\,74\pm 3,\,60\pm 3]$ degrees for the left, center and right positions, respectively.

To estimate the arm’s endpoint stiffness, it is necessary to physically perturb the arm’s position from the resting position **x**_0_. This was accomplished by a small rapid perturbation, corresponding to a fifth order polynomial of the form

2 $${{\bf{x}}}_{p}(t)=[\begin{array}{c}\frac{30A}{{T}^{5}}(\frac{{T}^{2}}{3}{t}^{3}-\frac{T}{2}{t}^{4}+\frac{1}{5}{t}^{5})\\ 0\end{array}]\,{\rm{for}}\,0\le t\le T$$

where $A=7$ millimetres and *T* = 100 milliseconds. The position perturbation started from zero when *t* = 0 and reached an amplitude *A* when *t* = *T*.

The position perturbation **x**_*p*_(*t*) was rotated by a rotation matrix ${\bf{R}}(\varnothing )$ with rotation angles selected from the array $\varnothing =[0,\,45,\,90,\,135,\,180,\,225,\,270,\,315]$ degrees, and was added to the setpoint command **x**_0_ to perturb the arm’s position in eight different directions. After the 100-millisecond position perturbation ramp, the perturbation ${{\bf{x}}}_{p}(t)={[A,0]}^{T}$ was held for 400 milliseconds, after which it ramped back down to zero in another 100 milliseconds.

Figure 1B shows two sample position perturbations along the *x*-axis. In this plot, $dx=x(t)-{x}_{0}$ and $d{F}_{x}={F}_{x}(t)-{F}_{{x}_{0}}$. The top panel shows how the position rapidly changed to the setpoint position in 150 milliseconds, held it there steadily for 400 milliseconds before ramping back down to *dx* = 0. The shaded areas denote where the restoring force was measured. The velocity of the endpoint in the shaded area was effectively null. All force data was low-pass filtered using a zero-lag, second-order Butterworth filter with a cutoff frequency of 20 Hz. The change in the filtered force signal along the *x*-axis *dF*_*x*_, which is plotted in the bottom panel of Fig. 1B, exhibited a constant non-zero value when the perturbation was stable and maximal in the shaded areas of Fig. 1B. This non-zero steady-state value arose from the endpoint stiffness.

### Experimental protocol

Prior to the start of the experiment, the experimenter instructed the subject to maintain a specific level of grasp force as shown on the monitor. A red circle displayed the target grasp force that subjects had to produce and maintain. The applied grasp force was displayed by a filled white circle that grew in size. Once the subject held the target grasp force within a tolerance of ±1 N for 300 milliseconds and was applying less than 1.5 N of load force against the robot, the robot began to perturb the subject’s arm in eight directions chosen at a random order. Subjects were instructed to ignore the position perturbations and focus only on maintaining the target grasp force. During the position perturbation period, which lasted 600 milliseconds, the feedback of the hand’s position and the applied grasp force was frozen to the last value measured prior to the position perturbation. This ensured that subjects did not exhibit correctional movements driven by visual feedback during the perturbation^15^. The visual feedback was restored between each perturbation. Once the eight perturbations were finished, subjects were instructed to relax for 5 seconds, after which another target grasp force was presented.

The target grasp force, which ranged from [5, 10, 15, 20] N, was selected in a random order. The order in which the endpoint stiffness was measured for the three different postures was fixed for all subjects. First, subjects matched all four grasp force levels in the left posture, and then the center posture, and finally the right posture. This process was repeated again for a total 3 cycles such that each posture and each grasp force level was tested three times to have a robust estimate of the endpoint stiffness, and to assess the consistency of our measurements. Hence, 24 perturbations were measured at each posture and each grasp force level per subject.

### Estimation of endpoint stiffness

For small perturbations, we assumed that the inertia **M**, viscosity **D** and stiffness **K** of the arm were linear^7^ such that the dynamics could be described by

3 $${\bf{M}}\cdot {\bf{d}}\ddot{{\bf{x}}}+{\bf{D}}\cdot {\bf{d}}\dot{{\bf{x}}}+{\bf{K}}\cdot {\bf{dx}}=-\,{\bf{dF}}$$

where ${\bf{d}}{\bf{x}}={\bf{x}}(t)-{{\bf{x}}}_{0}$, ${\bf{d}}{\bf{F}}={\bf{F}}(t)-{{\bf{F}}}_{0}$ and **F**_0_ is the force measured prior to the perturbation. Once the position of the arm reaches its perturbed steady-state value with null velocity and null acceleration, this equation simplifies to

4 $${\bf{K}}\cdot {\bf{dx}}=-\,{\bf{dF}}.$$

The quantities of **dx** and **dF** were measured by the KINARM’s position sensing and its six-axis force sensor. This data was linearly regressed to find the endpoint stiffness **K**. The data from all perturbations in all trials was used in the analysis to estimate the endpoint stiffness for a given posture and grasp force level per subject.

For a specific grasp force level and posture, we gathered the data from the 100-millisecond perturbation window (refer to the shaded regions in Fig. 1B) from all eight directions and all three measurements per direction. The perturbation window was approximately 200 milliseconds after the end of the position perturbation, where voluntary muscle activity does not contribute to the restoring force^13^.

The endpoint stiffness matrix **K** can be visualized as an ellipse of restoring forces that is produced when a unit position perturbation vector is multiplied by the endpoint stiffness matrix^13^. The unit perturbation vector is slowly rotated in increments between the angles of zero and 360 degrees to calculate the restoring force generated by the endpoint stiffness. By plotting these restoring forces, an ellipse is generated whose size and orientation reveal in which direction the restoring force is greatest. The larger the ellipse, the larger the restoring force. The major axis of the ellipse is the direction in which the endpoint stiffness is greatest. The size of the endpoint stiffness was computed by the product of the length of the major and minor axes of the endpoint stiffness ellipse with π.

### Measurement of muscle activity

In the second experiment, 10 additional participants were recruited to measure the muscular activity in the elbow and shoulder while grasping the robotic interface. In this experiment, we only measured the stiffness of the arm in the center posture, and tested each grasp force level 5 times. The electromyography (EMG) was measured using the Delsys Trigno wireless EMG sensor system at 1000 Hz from the elbow monoarticular pair (brachioradialis and triceps lateral head); the biarticular pair (biceps brachii and the triceps long head); and the shoulder muscle pairs were measured (pectoralis major and the posterior deltoid). In this experiment, subjects also wore a wrist brace to prevent wrist movements.The mean EMG activity in the same 100-millisecond window, where the restoring forces were measured, was calculated for each perturbation.

The arm’s joint stiffness matrix is composed of the stiffness of each muscle in the upper arm, giving

5 $$\begin{array}{ccc}{{\bf{K}}}_{j} & = & [\begin{array}{cc}{K}_{s}+{K}_{b} & {K}_{b}\\ {K}_{b} & {K}_{e}+{K}_{b}\end{array}]\\  & = & [\begin{array}{cc}{K}_{s+}+{K}_{s-}+{K}_{b+}+{K}_{b-} & {K}_{b+}+{K}_{b-}\\ {K}_{b+}+{K}_{b-} & {K}_{e+}+{K}_{e-}+{K}_{b+}+{K}_{b-}\end{array}]\end{array}$$

where the subscript *s*, *b* and *e* denote the shoulder, the biarticular and the elbow, and the positive and negative subscripts indicate the flexor and extensor muscle, respectively. Under the Kelvin-Voigt muscle model, the stiffness of a muscle is proportional to its muscle activation^16–18^, e.g. ${K}_{s+}\propto {u}_{s+}$. Thus, the terms in the joint stiffness matrix are related to the muscle activities in the arm by

6 $${{\bf{K}}}_{j}\propto [\begin{array}{cc}{u}_{s}+{u}_{b} & {u}_{b}\\ {u}_{b} & {u}_{e}+{u}_{b}\end{array}]$$

where ${u}_{s}={u}_{s+}+{u}_{s-}$ is the summed muscle activity of the shoulder flexor-extensor, *u*_*b*_ is the sum of the biarticular activity and *u*_*e*_ is the sum of the elbow monoarticular muscle pair’s activity. Hence, an increase in the activity of the elbow flexor and/or extensor with greater grasp force would imply an increase in the stiffness of the elbow joint.

To compare the activation of the muscles between subjects, the activity of each muscle was normalized by dividing it with its average activity in all trials,

7 $$U=\frac{u}{{\bar{u}}_{\varnothing jk}}$$

where the index $\varnothing$ denotes the angle of the perturbation (of a total eight directions), *j* is the repeated measurement index (of a total five repetitions) and *k* is the grasp force level (of a total four levels). Altogether, the averaged activity ${\bar{u}}_{\varnothing jk}$ would be the measured activity in 160 measurements (=8 × 5 × 4). This was carried out for every muscle in every subject to compare the changes in the muscle activity as a function of the grasp force between subjects.

### Statistical testing

We used a two-way repeated-measures analysis of variance to examine the effect of the grasp force and the posture on the size of the endpoint stiffness ellipse. One-sample t-tests with a Holm-Bonferoni correction were conducted on the normalized EMG activity of the elbow, shoulder and cross-joint to find the significance of the linear gradient between the EMG data and the grasp force.

## Results

Figure 1B shows the position, velocity and restoring force along the *x*-axis from a sample trial containing two position perturbations. The shaded regions in Fig. 1B are the windows where the data are measured. To ensure that the arm’s viscosity and inertia did not contribute to the measurement of the restoring force, we calculated the endpoint’s velocity during the 100-millisecond window where the restoring force was measured for all subjects. The endpoint’s group mean velocity was found to be 0.043 ± 0.005 cm/s (mean and standard error), which confirmed that the arm was effectively stationary during this phase of the perturbation. Figure 1B also shows that enough time was set between each perturbation for the arm to come to rest to its original position *dx* = 0. For convenience, all subsequent values with errors bars will denote the group mean and the standard error.

We examined if the subjects were successful in maintaining the target grasp force during the experiment. The grasp force was averaged in a 100-millisecond window of the perturbation near the end of the position perturbation. This average grasp force was calculated per perturbation direction, and the mean of all directions and postures was taken to find the group mean grasp force for each target level of grasp force. The group mean grasp force, along with the standard error, were found to be 4.79 ± 0.06 N, 9.55 ± 0.09 N, 14.30 ± 0.10 N and 19.14 ± 0.10 N. The grasp force was comfortably within a tolerance of 4% of the target level, implying that the subjects were successful in maintaining the grasp force during the perturbations.

Next, we looked at the restoring force in the left, center and right positions from a sample subject (see Fig. 1C). The lighter colors denote a stronger grasp force. The lines connect the three repeated measurements for each position perturbation direction. The restoring force was symmetrically distributed around the origin. The restoring force from the stronger grasp force trials was larger than during a weaker grasp. This restoring force data was linearly regressed with the position perturbation data to estimate the endpoint stiffness at each grasp force level, separately for each posture.

The endpoint stiffness from the same sample subject is drawn as ellipses from the left, center and right postures in Fig. 1D. The major axes of the stiffness ellipses was pointed towards the subject’s shoulder, resembling the endpoint stiffness observed in other studies^6,7,19^. To confirm the goodness-of-fit, we calculated the coefficient of determination, or the amount of variance explained by our linear regression model for each posture and each grasp force level per subject. The group mean was R^2^ = 0.88 ± 0.02, implying that 88% of the variance in the restoring force could be explained by the fitted endpoint stiffness values, demonstrating that our experimental methodology yielded consistent data.

**Figure 2:**
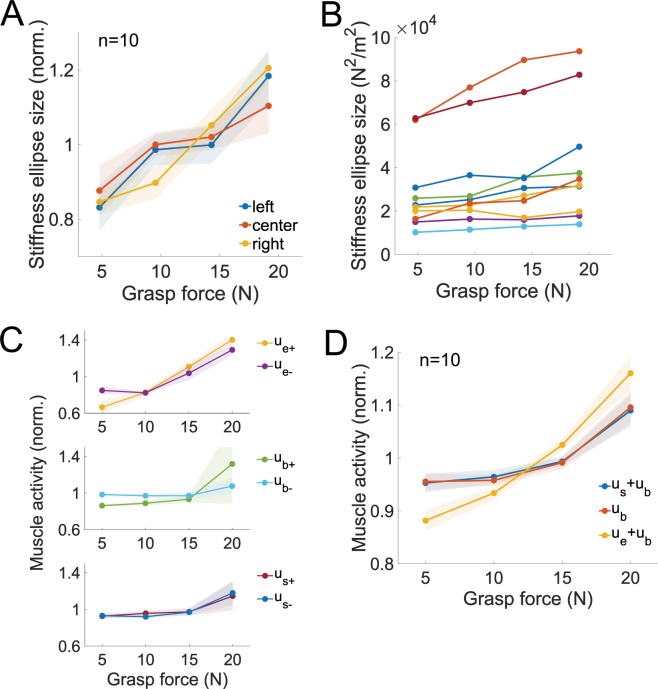
The arm’s endpoint stiffness increased with the grasp force irrespective of posture. The co-contraction of the arm’s muscles was observed to increase with the grasp force. (**A**) The normalized size of the stiffness ellipse is plotted as a function of the grasp force for the left (blue), center (red) and right (yellow) positions. The size of the stiffness ellipse increased linearly with grasp force. Importantly, the arm’s posture or position in the workspace had no effect on the relationship between the arm’s stiffness and the grasp force. (**B**) The size of the endpoint stiffness ellipse is plotted as a function of the grasp force for every subject, denoted by a different color. The data from the three postures were averaged to generate this plot. The size of the stiffness ellipse was in the range reported in previous studies. (**C**) The normalized muscle activity from the six muscles in the arm are plotted as a function of the grasp force. The data come from a single subject with the shaded area showing one standard deviation. The activity of the monoarticular elbow, biarticular and shoulder muscles increased with the grasp force. (**D**) The group mean normalized activity of the six muscles measured in the arm are plotted as a function of the grasp force, with the shaded region representing one standard error. The activation *u*_s_ was calculated by taking the sum of the flexor and extensor activities *u*_s+_ and *u*_s-_ respectively, and was renormalized for between-subject comparisons. This data was linearly regressed for each subject, and statistical testing showed that the muscle activity in the elbow, shoulder and cross-joints all significantly increased as a function of the grasp force.

Table 1 shows the group mean and standard error values of the two-dimensional endpoint stiffness matrix for the left, center and right positions at all grasp force levels. From these estimates of the endpoint stiffness matrix, we calculated the size of the endpoint stiffness ellipses for every subject at every grasp force level and at every posture. For each subject and posture, the stiffness ellipse was normalized by dividing the mean size of the ellipse in all grasp force levels for between-subject comparisons. The group mean normalized size of the stiffness ellipses are plotted as a function of the group mean grasp force in Fig. 2A. Contrary to our hypothesis, a two-way repeated-measures ANOVA found no significant interaction between the posture and grasp force in explaining the size of the stiffness ellipse (*F*(6, 54) = 0.81, *p* > 0.57). On the other hand, the repeated-measures ANOVA revealed a significant effect of the grasp force on the size of the endpoint stiffness ellipse (*F*(3, 54) = 43.5, *p* < 10^−9^), supporting our hypothesis that the grasp force is positively correlated with the magnitude of the endpoint stiffness. The normalized size of the stiffness ellipse grew by 37% when the grasp force increased from 5 N to 20 N (normalized ellipse size increased from 0.85 ± 0.03 to 1.16 ± 0.04). We also calculated the size of the endpoint stiffness ellipse for each subject as a function of the grasp force, averaging the data over the three different positions. Figure 2B shows the size of the endpoint ellipse for each subject, which was found to be between 10^4^ N^2^/m^2^ and 10^5^ N^2^/m^2^ for our ten subjects, resembling the range observed in other studies^6,7^.

**Table 1:** The group mean and standard error of the endpoint stiffness matrix parameters **K**_*xx*_, **K**_*xy*_, **K**_*yx*_ and **K**_*yy*_ in the left, center and right positions are shown for the different levels of grasp force.

Stiffness (N/m)	Grasp force			
	5 N	10 N	15 N	20 N
K_*xx*_ (left)	90.0 ± 9.5	92.3 ± 8.5	95.1 ± 10.7	99.5 ± 9.2
K_*xy*_ (left)	−78.1 ± 7.9	−81.4 ± 8.9	−83.6 ± 10.4	−86.6 ± 9.6
K_*yx*_ (left)	−66.5 ± 8.3	−65.6 ± 7.6	−72.0 ± 11.6	−72.8 ± 7.8
K_*yy*_ (left)	151.9 ± 17.4	166.7 ± 21.1	173.6 ± 22.8	189.1 ± 25.9
K_*xx*_ (center)	49.2 ± 6.2	52.1 ± 6.6	52.0 ± 7.6	52.7 ± 7.0
K_*xy*_ (center)	−14.5 ± 2.4	−14.1 ± 5.0	−12.9 ± 3.4	−12.9 ± 3.8
K_*yx*_ (center)	−12.2 ± 4.6	−5.1 ± 5.9	−3.1 ± 5.7	−1.3 ± 4.8
K_*yy*_ (center)	166.9 ± 20.3	181.9 ± 22.7	191.6 ± 25.2	205.2 ± 27.2
K_*xx*_ (right)	73.3 ± 6.4	74.3 ± 8.0	81.3 ± 8.4	87.0 ± 9.7
K_*xy*_ (right)	48.7 ± 10.6	57.2 ± 12.6	54.5 ± 14.1	57.2 ± 15.3
K_*yx*_ (right)	58.8 ± 13.3	63.9 ± 13.0	66.9 ± 15.9	68.1 ± 17.0
K_*yy*_ (right)	175.6 ± 17.1	191.9 ± 19.6	202.0 ± 22.9	212.5 ± 23.1

What caused the endpoint stiffness of the arm to increase with the grasp force? We recruited a new set of 10 subjects and measured their endpoint stiffness as a function of the grasp force. In this experiment, six wireless surface electromyography (EMG) sensors were placed on the subject’s elbow monoarticular, biarticular and shoulder muscle pairs (see Methods for selected muscles). The mean muscle activity was measured in the 100-millisecond window where the restoring force from the arm was measured. For this experiment, we only measured the endpoint stiffness in the center position, and each grasp force level was measured five times. Subjects also wore a wrist brace to prevent wrist movements. The activity of each muscle was normalized by dividing its mean activity measured in all grasp force levels (see Methods for details).

Table 2 shows the group mean and standard error endpoint stiffness values for all grasp force levels. As with the data shown in Table 1 from the previous experiment, the endpoint stiffness values increased with the grasp force.

**Table 2:** The group mean and standard error of the endpoint stiffness matrix parameters **K**_*xx*_, **K**_*xy*_, **K**_*yx*_ and **K**_*yy*_ in the center position from the second set of 10 subjects where the EMG from the six muscles in the arm were also measured.

Stiffness (N/m)	Grasp force			
	5 N	10 N	15 N	20 N
K_*xx*_ (center)	45.0 ± 5.4	48.4 ± 5.6	51.6 ± 7.3	58.1 ± 10.7
K_*xy*_ (center)	−22.7 ± 2.7	−25.9 ± 4.5	−24.5 ± 4.1	−26.7 ± 5.2
K_*yx*_ (center)	−11.2 ± 4.8	−12.8 ± 6.5	−7.5 ± 5.0	−11.1 ± 6.2
K_*yy*_ (center)	129.2 ± 12.0	140.8 ± 9.7	154.7 ± 17.5	167.6 ± 21.8

The normalized muscle activity from the elbow, biarticular and shoulder muscle pairs are plotted as a function of the grasp force from a sample subject in Fig. 2C. The summed muscle activity of the shoulder muscle pair *u*_s_ and the biarticular pair *u*_b_ contribute to the shoulder joint stiffness *K*_ss_, and the elbow joint stiffness *K*_ee_ is composed of the activity of the elbow monoarticular pair *u*_e_ and the biarticular pair *u*_b_, whilst the activity of the biarticular pair *u*_b_ contributes to the cross-joint stiffness *K*_se_ and *K*_*e*s_. The sum of the normalized activities of these muscles is plotted as a function of grasp force in Fig. 2D. We linearly regressed the three curves for each subject to examine the significance of their gradients. Using one-sample t-tests with a Holm-Bonferoni correction, the muscle activity that contributed to the shoulder joint stiffness (*t*(9) = 2.87, *p* < 0.019), the elbow joint stiffness (*t*(9) = 5.86, *p* < 0.002) and the cross-joint stiffness (*t*(9) = 3.41, *p* < 0.008) significantly increased with the grasp force. Thus, the co-contraction in the elbow, shoulder and the cross-joint contributed to the increase in the magnitude of the arm’s endpoint stiffness.

## Discussion

We perturbed the position of the arm to estimate the arm’s endpoint stiffness in three different positions of the workspace with different levels of power grasp force. The magnitude of the endpoint stiffness increased with the grasp force. This relationship was observed in the three different postures that we tested, implying that the linear correlation between the grasp force and the endpoint stiffness magnitude was robust under different postures.

The restoring force was symmetrically distributed around the origin in the left, center and right positions, implying that for small position perturbations and a minimum grasp force of 5 N, there is little risk of the robotic handle slipping out of the subject’s hand, which may adversely influence the measurement of the endpoint stiffness. Furthermore, the high proportion of the variance explained by the linear regression of the restoring force against the position perturbation shows that a consistent estimate of endpoint stiffness was obtained by our perturbation methodology where visual feedback of the grasp force was provided.

Our study cannot determine whether the primary contributor to the increase in the endpoint stiffness was the grasp stiffness or the stiffness of the arm. However, the EMG data shows that the increase in the endpoint stiffness was not solely due to greater grasp stiffness, which is known to increase with the grasp force^20^. By measuring the muscle activity of the elbow monoarticular pair, the biarticular pair and the shoulder pair in the second experiment, we found that muscular co-contraction of the elbow, shoulder and cross-joints increased with the grasp force. This synergistic activation has been previously observed between the power grasp and either the elbow or the shoulder muscles in other studies^21,22^. The increase in muscle activity was somewhat more pronounced in the elbow monoarticular pair in comparison to the biarticular and the shoulder muscle pair. This suggests that muscles closer to the hand activate more strongly during grasping in comparison to muscles further away from the hand.

In another study, we found that the grasp force increased when subjects desired higher movement precision in two reaching tasks^11^. The reaching tasks tested in this study were inspired by earlier studies that had reported an increase in endpoint stiffness during their tasks^3–5^. In one of these tasks, subjects reached in an unstable force field that pushed the hand left or right when it deviated from the midline. This task required higher endpoint stiffness to precisely move along the midline to reduce the effects of the unstable force field^4^. The grasp force measured during the learning of this task^11^ resembled the change in the co-contraction of the arm reported in another study^23^. Namely, the grasp force increased rapidly over the course of five trials, and then exponentially declined in magnitude and plateaued at a level higher than in training trials without the force field.

Although the grasp force methodology may signify changes in the magnitude of the arm’s endpoint stiffness, the spatial information of the endpoint stiffness is lost. The grasp force is unsuitable for measuring a change in the orientation of the stiffness ellipse, which might be useful when examining how humans optimize their endpoint stiffness to counter unstable or unpredictable environments^4^. The loss of the stiffness ellipse’s orientation is traded off by the superior temporal information provided by the grasp force. As was observed in a previous study of ours^11^, the grasp force changed not only between trials, but within each reaching movement. The change in the grasp force resembled the temporal profile of the movement imprecision when reaching in an unstable force field. EMG also provides a high temporal resolution estimate of the endpoint stiffness of the arm, but it requires calibration at different postures to be effective in estimating the endpoint stiffness during reaching^19^. The grasp force, on the other hand, was constant during normal reaching and was not influenced by the load force nor by external forces imposed by a robotic interface^11^. Thus, analyzing the changes in the grasp force may further our understanding of the adaptation in endpoint stiffness during both postural and reaching tasks.

## References

[1] Hogan N (1984). Adaptive control of mechanical impedance by coactivation of antagonist muscles. IEEE Trans. Autom. Control.

[2] Selen LPJ, Franklin DW, Wolpert DM (2009). Impedance Control Reduces Instability That Arises from Motor Noise. J. Neurosci..

[3] Wong J, Wilson ET, Malfait N, Gribble PL (2009). Limb Stiffness Is Modulated With Spatial Accuracy Requirements During Movement in the Absence of Destabilizing Forces. J. Neurophysiol..

[4] Burdet E, Osu R, Franklin DW, Milner TE, Kawato M (2001). The central nervous system stabilizes unstable dynamics by learning optimal impedance. Nature.

[5] Franklin DW (2007). Endpoint Stiffness of the Arm Is Directionally Tuned to Instability in the Environment. J. Neurosci..

[6] Flash T, Mussa-Ivaldi F (1990). Human arm stiffness characteristics during the maintenance of posture. Exp. Brain Res..

[7] Tsuji T, Morasso PG, Goto K, Ito K (1995). Human hand impedance characteristics during maintained posture. Biol. Cybern..

[8] Perreault EJ, Kirsch RF, Acosta AM (1999). Multiple-input, multiple-output system identification for characterization of limb stiffness dynamics. Biol. Cybern..

[9] Burdet E (2000). A method for measuring endpoint stiffness during multi-joint arm movements. J. Biomech..

[10] Piovesan D, Pierobon A, DiZio P, Lackner JR (2013). Experimental measure of arm stiffness during single reaching movements with a time-frequency analysis. J. Neurophysiol..

[11] Takagi A, Kambara H, Koike Y (2019). Increase in Grasp Force Reflects a Desire to Improve Movement Precision. eNeuro..

[12] Franklin DW (2008). CNS Learns Stable, Accurate, and Efficient Movements Using a Simple Algorithm. J. Neurosci..

[13] Mussa-Ivaldi FA, Hogan N, Bizzi E (1985). Neural, mechanical, and geometric factors subserving arm posture in humans. J. Neurosci..

[14] Darainy M, Malfait N, Gribble PL, Towhidkhah F, Ostry DJ (2004). Learning to Control Arm Stiffness Under Static Conditions. J. Neurophysiol..

[15] Sarlegna F (2003). Target and hand position information in the online control of goal-directed arm movements. Exp. Brain Res..

[16] Katayama M, Kawato M (1993). Virtual trajectory and stiffness ellipse during multijoint arm movement predicted by neural inverse models. Biol. Cybern..

[17] Osu R, Gomi H (1999). Multijoint Muscle Regulation Mechanisms Examined by Measured Human Arm Stiffness and EMG Signals. J. Neurophysiol..

[18] Shin D, Kim J, Koike Y (2009). A Myokinetic Arm Model for Estimating Joint Torque and Stiffness From EMG Signals During Maintained Posture. J. Neurophysiol..

[19] Gomi H, Kawato M (1997). Human arm stiffness and equilibrium-point trajectory during multi-joint movement. Biol. Cybern..

[20] Höppner H, McIntyre J, Smagt P (2013). van der. Task Dependency of Grip Stiffness—A Study of Human Grip Force and Grip Stiffness Dependency during Two Different Tasks with Same Grip Forces. PLOS ONE.

[21] Miller LC, Dewald JPA (2012). Involuntary paretic wrist/finger flexion forces and EMG increase with shoulder abduction load in individuals with chronic stroke. Clin. Neurophysiol..

[22] Sporrong H, Palmerud G, Herberts P (1996). Hand grip increases shoulder muscle activity, An EMG analysis with static hand contractions in 9 subjects. Acta Orthop. Scand..

[23] Franklin DW, Osu R, Burdet E, Kawato M, Milner TE (2003). Adaptation to Stable and Unstable Dynamics Achieved By Combined Impedance Control and Inverse Dynamics Model. J. Neurophysiol..

